# Degradation Mechanism of Coal Gangue Concrete Suffering from Sulfate Attack in the Mine Environment

**DOI:** 10.3390/ma16031234

**Published:** 2023-01-31

**Authors:** Linli Yu, Junwu Xia, Jixin Gu, Shuai Zhang, Yu Zhou

**Affiliations:** 1 State Key Laboratory for Geomechanics and Deep Underground Engineering, China University of Mining and Technology, Xuzhou 221116, China; 2 Jiangsu Collaborative Innovation Center of Building Energy-Saving and Construction Technology, Jiangsu Vocational Institute of Architectural Technology, Xuzhou 221116, China

**Keywords:** coal gangue concrete, mine environment, sulfate attack, compressive strength, microscopic degradation mechanism

## Abstract

Recycling coal gangue as aggregate to produce concrete in situ is the most effective way to solve the problem of deposited coal gangue in mines. Nevertheless, the mine environment underground is rich in sulfate ions, posing a threat to the durability of coal gangue concrete (CGC). Hence, the degradation process of sulfate-attacked CGC is investigated. A series of tests is performed to evaluate its variation law of mass, dynamic elastic modulus, compressive strength and sulfate ion distribution. Meanwhile, the microstructure and phases of sulfate-attacked CGC are identified by scanning electron microscopy, X-ray diffraction and thermogravimetric analysis. The results indicate that the residual compressive strength ratio of CGC is higher than that of normal concrete after a 240 d sulfate attack, implying a superior sulfate resistance for CGC. Additionally, the higher the sulfate concentration, the more severe the degradation. Except for the secondary hydration of CGC itself, the diffused sulfate ions also react with Ca(OH)_2_, forming gypsum and ettringite; this plays a positive role in filling the pores at the early stage, whereas, at the later stage, the generated micro-cracks are detrimental to the performance of CGC. In particular, the proposed sulfate corrosion model elucidates the degradation mechanism of CGC exposed to a sulfate-rich environment.

## 1. Introduction

Coal gangue is solid waste generated from coal excavating and processing. In China, deposited coal gangue amounts to about 7 billion tons and increases at a rate of 200–350 million tons per year, leading to vast land occupation and severe environmental pollution in mines [1,2,3]. Thus, the eco-friendly utilization of coal gangue becomes an urgent issue. Extensive research has demonstrated that coal gangue can be effectively used as a substitute for partial cementitious powder, fine or coarse aggregate in concrete, which not only alleviates the problem of deposited coal gangue but also reduces the exploitation of river sand and natural gravel [4]. Yu, et al. [5] used coal gangue to entirely replace fine and coarse aggregate, preparing coal gangue concrete (CGC) of C30–C50 grade. Gong, et al. [6] discovered that the gangue concrete exhibited a certain post-peak carrying capacity, applicable for backfilling gob-side entry retaining in mine construction. Xiao, et al. [7,8] and Chen, et al. [9] proposed that CGC could be used locally as a shotcrete for coal mine tunnels underground, with the workability and strength meeting the requirements of some coal mining engineering. Obviously, recycling coal gangue to produce concrete in situ is the most economic and effective way.

However, the in situ environment, especially the mine water, includes various chemical compositions, particularly a high content of SO_4_^2−^ (even 1500–2000 mg/L), which may cause deterioration of cementitious materials and concrete structures in underground mining areas [10,11,12,13]. Wang, et al. [10] elucidated the compressive properties’ evolution of engineered cementitious composites under the attack of high sulfate coal mine water, and the strength was reduced at the later corrosion stage. Moreover, it is claimed that sulfate attack has a great impact on the pre- and post-failure behavior of cemented paste backfill in underground mines [11]. The promotion of the use of CGC is affected by its strength and durability in a mine environment.

The performance of normal concrete (NC) exposed to sulfate attack has been extensively investigated. The typical characteristics, expansive cracks and spalling of sulfate-attacked concrete, were commonly observed, accompanied by volume expansion and mass loss [14]. Zhao, et al. [15] discovered that the mass of concrete was reduced by approximately 10% after being immersed in a 5% sulfate solution for 9 months. In terms of mechanical properties, many studies have paid much attention to the compressive strength deterioration of concrete in a sulfate environment, considering variables such as water-to-cement ratio [16], additives [17], sulfate concentration [18], dry-wet cycle [19] and erosion age [20]. Moreover, the compressive strength of sulfate-attacked concrete mostly exhibited a first rising and then dropping trend [15,16]. This is attributed to the complicated physical and chemical processes during sulfate attack, including mainly sulfate crystallization and sulfate corrosive reaction [21]. More importantly, a chemical reaction occurred between the diffused sulfate ions and the hydrated products of cement, forming the expansive products of gypsum and ettringite, which would affect the performance of concrete [22,23,24]. Peng, et al. [20,25] conducted a series of micro tests, including X-ray diffraction (XRD), SEM, Fourier transform infrared spectroscopy (FTIR) and thermogravimetric (TG) analysis, to figure out the deterioration mechanism of sulfate-attacked concrete and demonstrated that the main sulfate products are gypsum and ettringite. As discussed above, the performance and micro products of NC were significantly affected by the sulfate-rich environment. Nevertheless, for sulfate-attacked CGC, the corrosion process and degradation mechanism are still not clear. The activated SiO_2_ and Al_2_O_3_ of coal gangue could participate in the hydrated reaction of CGC, different from that of NC [26,27]. Wang, et al. [28] discovered that concrete using waste fly ash and coal gangue is feasible for application in coal mining subsidence areas with high groundwater levels. Tang, et al. [29] used coal gangue to replace the cement of sustainable concrete, which exhibited a positive effect on sulfate resistance. Moreover, similar results were obtained by Ma, et al. [30]. Hence, to expand the scale of utilization of CGC in situ, it is necessary to investigate its degradation properties and mechanisms when suffering from sulfate attack in the mine environment.

In this paper, to investigate the degradation process and mechanism of CGC caused by sulfate attack, green CGC with all-coal-gangue aggregate was prepared and then exposed to sulfate spray to simulate the mine water environment. Taking the CGC strength grade, sulfate concentration and corrosion time as variables, the variation law of mass, relative dynamic elastic modulus and compressive strength in different sulfate-attacked CGC were evaluated through a series of physical tests. Moreover, the concentration of sulfate ions at different depths was determined using the chemical titration method. After that, the microstructure and mineral phase of sulfate-attacked CGC were characterized by SEM, XRD and TG micro tests. Furthermore, a microscopic sulfate corrosion model of CGC was proposed to illustrate its degradation mechanism, providing a basis for the utilization of coal gangue in concrete exposed to a sulfate-rich environment.

## 2. Experimental Program

### 2.1. Materials

**Figure 1:**
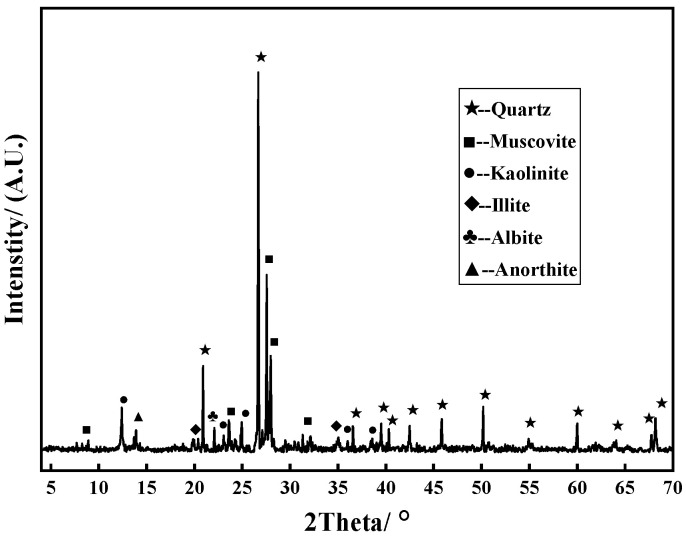
XRD pattern of coal gangue.

Ordinary Portland cement (CUCC, P.O 42.5R) was used as the binder material in this test. It has a specific surface area of 346 m^2^/kg and a density of 3.1 g/cm^3^, in compliance with the Chinese GB175-2007 standard. Coal gangue was provided by the Zhang Shuang Lou mine in Xuzhou, Jiangsu Province, China. The chemical composition was determined via X-ray fluorescence; as listed in Table 1, it was mainly SiO_2_ (61.69%) and Al_2_O_3_ (19.11%). Additionally, based on the results of X-ray diffraction (XRD), the XRD pattern in Figure 1 shows that the primary mineral components of the coal gangue were quartz and muscovite. With a jaw crusher and vibrated screen, the coal gangue was crushed and screened into fine and coarse aggregates for CGC. Simultaneously, river sand and natural gravel bought from a local supplier were used for NC as a contrast. The detailed properties of the aggregate are summarized in Table 2. Additionally, a superplasticizer (Dalian Sika Building Materials Co., Ltd, Dalian, China) was employed as a water reducer. Clean tap water from the laboratory was adopted to prepare the concrete samples and corrosive solutions. In addition, sodium sulfate (Na_2_SO_4_), with a purity of more than 99%, was purchased to prepare the corrosive sulfate solutions.

### 2.2. Sample Preparation

In the present study, three variables, including CGC strength grade (CGC20, CGC30 and CGC40), the sulfate concentration of the solution (S0, S5, S10 and S15) and corrosion time (28 d, 60 d, 90 d, 120 d, 180 d and 240 d), were considered to investigate the degradation mechanism of sulfate-attacked CGC. As a contrast, NC with a similar mix formulation as CGC30 was also considered. S0, S5, S10 and S15 refer to 0, 5%, 10% and 15% sulfate corrosive solutions in mass fraction, respectively. The “d” in “28 d, 60 d, 90 d, 120 d, 180 d and 240 d” represents days as a unit of time, and the “s” in “180 s, 120 s” stands for the seconds as a unit of time. According to the single-factor control method, the concrete with CGC30 or S10 was used as a benchmark, and Table 3 lists the detailed information for the test design. For each group, six parallel specimens were prepared for the following sulfate corrosive tests.

Based on previous trial results, three strength grades of CGC were prepared, with a group of NC for comparison. The four mix proportions are presented in Table 4. It is worth noting that the fine and coarse aggregates of CGC were totally replaced by coal gangue aggregates, to maximize the utilization of coal gangue in mines. For producing concrete, the materials and molds were prepared in advance. According to the test design and mix proportion, the weighed aggregates and cement were mixed in a mixer for 180 s, and then the water containing the corresponding superplasticizer was added gradually and blended for another 120 s. Subsequently, the fresh mixtures were cast into the cubic molds with a size of 100 mm × 100 mm × 100 mm and consolidated on the vibrating table. Finally, the specimens were demolded after 24 h and cured for 28 days in a curing room with a temperature of 20 ± 2 °C and a relative humidity of 95 ± 5%. Afterwards, the specimens were taken out and placed into corresponding corrosive conditions.

### 2.3. Corrosive Sulfate Conditions

Considering the high-sulfate nature of underground mine water, the sodium sulfate solution was selected to represent the chemical constituents of coal mine water in the present study. Circular spray tests were used to simulate sulfate-rich mine water and high humidity conditions in underground mines. According to the test design, Na_2_SO_4_ solutions with different concentrations were prepared in tanks in advance. The Na_2_SO_4_ solution was pumped into a pipe and then sprayed by spray headers onto the surface of the concrete specimens, where the specimens were placed in an enclosed space to ensure high relative humidity of about 90%. The specimens were sprayed twice a day, continuing for 120 s each time with a spraying speed of 0.003 m^3^/min for each spray header. It should be noted that the Na_2_SO_4_ solutions were checked and replenished weekly to ensure accurate sulfate concentration. Additionally, specimens exposed to tap water (S0) were set as a contrast. When the corrosive age was reached, the specimens were taken out from the Na_2_SO_4_ solution and subjected to the tests described below.

### 2.4. Testing Methods

#### 2.4.1. Physical and Mechanical Property Tests

(1) Mass change

The specimens were taken out from the sulfate exposure conditions upon reaching their corrosive age. Considering the water content of the concrete, the specimens were dried in the laboratory environment until the mass maintained a constant value before measuring. By Chinese standard GB/T50082-2012, the mass of the concrete specimen was determined by an electronic scale with an accuracy of 0.01 g. The mass change ratio of specimens was evaluated by the equation as follows:

(1) $$w_{t}=\frac{m_{t}-m_{0}}{m_{0}}\times 100\%$$

where *w_t_* is the mass change ratio of specimens; *m_t_* denotes the mass of the specimens at the sulfate corrosion time of *t*; *m*_0_ represents the initial mass of the specimens before the sulfate attack. The mass change ratio value of each group was averaged from six parallel specimens.

(2) Relative dynamic elastic modulus

The ultrasonic velocity of the specimens was measured by a non-metal ultrasonic detector (ZBL—U520), which is a non-destructive test method characterizing the inner micro-cracks of concrete. Two transducers with Vaseline were positioned in the middle of the opposing surface. A generated ultrasonic pulse was transmitted to the concrete surface by a transmitter transducer and then received by the receiver transducer on the opposite side after traveling through the concrete. All the specimens were tested three times to obtain the averaged ultrasonic velocity. The relative dynamic elastic modulus values of the concrete specimens were calculated according to the following equation:

(2) $$R_{dt}=\frac{E_{dt}}{E_{d0}}=\frac{{\left(v_{t}\right)}^{2}}{{\left(v_{0}\right)}^{2}}$$

where *R_dt_* is the relative dynamic elastic modulus value; *E_dt_* and *v_t_* are the relative dynamic elastic modulus and ultrasonic velocity of the specimens at the sulfate corrosion time of *t*, respectively; *E_d_*_0_ and *v*_0_ are the relative dynamic elastic modulus and ultrasonic velocity of the specimens before sulfate attack, respectively.

(3) Compressive strength

**Figure 2:**
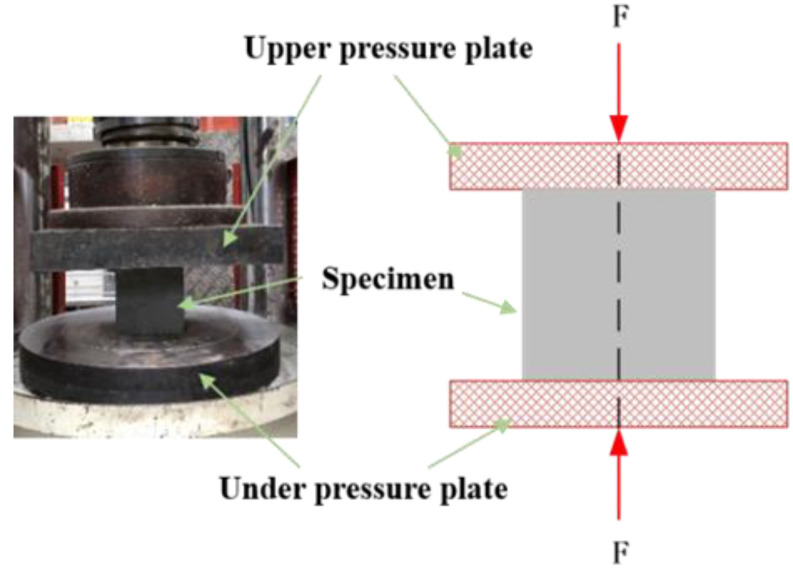
Compressive test of the cubic specimens.

According to the Chinese standard GB/T 50081–2002, a YAW-3000 hydraulic compression machine was employed to test the compressive strengths of the specimens, as shown in Figure 2. The cubic specimen was placed in the center of the upper and under loading plate to ensure the uniformity of applied pressure. After setting the section parameter of the specimen, the compressive test was conducted at a rate of 0.3 MPa/s, according to the Chinese standard GB/T 50081-2019. Meanwhile, the force and displacement were recorded by the data acquisition system of the testing machine. The compressive strengths were evaluated as follows:

(3) $$f_{c}=0.95\frac{F}{A}$$

where *f_c_* is the compressive strength of the cubic specimens (MPa); *F* is the peak load (N); and A is the compressed surface area (mm^2^). Additionally, the residual compressive strength ratio was proposed to elucidate the change after sulfate corrosion, and the equation was as follows:

(4) $$\beta_{t}=\frac{f_{ct}}{f_{c0}}$$

where $\beta_{t}$ is the residual compressive strength ratio; $f_{ct}$ denotes the compressive strength of the specimen at the sulfate corrosion time of *t*; and $f_{c0}$ is the corresponding compressive strength of the specimen before the sulfate stack. The values presented were averaged from the six parallel specimens with the error margin.

#### 2.4.2. Chemical Tests of Sulfate Concentration Distribution

**Figure 3:**
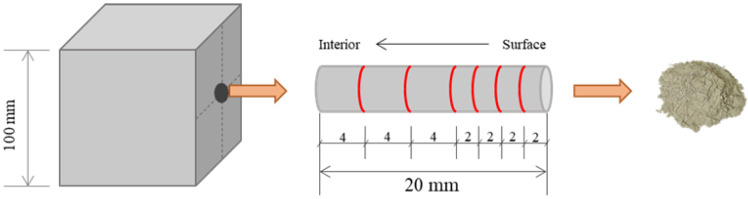
Diagram of powder sample preparation for sulfate ion concentration test.

After the exposure to sulfate corrosion, eight groups were selected to analyze their distribution of sulfate ions at different depths, considering different corrosion times, CGC strength grade and sulfate concentration of solution. A drill with different lengths was used to collect CGC powder samples at different depths. As shown in Figure 3, by drilling into a sulfate-attacked surface, the powder samples of seven different depth ranges were obtained for each group, including 0–2 mm, 2–4 mm, 4–6 mm, 6–8 mm, 8–12 mm, 12–16 mm and 16–20 mm. After being sieved through a 0.045 mm mesh and dried for 24 h at a temperature of 60 °C in a drying oven, the powder samples with different depth ranges were kept in sealed bags for the following sulfate concentration tests.

The content of sulfate ions in each powder sample was determined by chemical titration based on the Chinese standard GB/T 176-2017. The main operation steps were as follows: First, the powder sample was weighed by a scale with an accuracy of 0.0001 g and dissolved in hydrochloric acid to filter impurities. Then, hot barium chloride (BaCl_2_) was slowly added to the boiling solution to form a precipitation of barium sulfate–(BaSO_4_). After a long period of static settlement and filtration, the obtained barium sulfate was dried to constant mass. Finally, the filter membrane and dried precipitation of barium sulfate were weighed and recorded. The values of sulfate ion concentration at different depths were calculated by the equation as follows:

(5) $$\omega \left({\mathrm{SO}}_{4}^{2-}\right)=\frac{m_{2}-m_{1}}{m_{0}}\times 0.412\times 100\%$$

where $\omega \left({\mathrm{SO}}_{4}^{2-}\right)$ denotes the percentage of sulfate ion content; *m*_2_ is the total mass of barium sulfate and filter membrane; *m*_1_ is the mass of filter membrane; *m*_0_ is the mass of the weighed CGC powder samples; and 0.412 is the ratio of relative molecular mass between SO_4_^2−^ and BaSO_4_.

#### 2.4.3. SEM, XRD and TG tests

To clarify the micro degradation mechanism of CGC, nine specimens were chosen for SEM, XRD and TG tests. After the sulfate corrosion and compressive test, the appropriate fragments of each group were collected and immersed in ethanol solution for 7 d to terminate the hydration. Then, they were dried in a vacuum oven at 60 °C for 48 h. Suitable fragments at a depth of 2 mm from the surface were selected as samples for SEM, and the ground powder samples (200 mesh) with a depth of 0–5 mm were prepared for XRD and TG tests.

SEM observation was performed to investigate the micromorphology of the hydrated products after sulfate attack. The dried specimens were coated with gold conductive film and then observed by the SEM Quanta 250 (America FEI Company, Portland, America). Additionally, an XRD (D8 ADVANCE, Germany Bruker AXS Co., Ltd, Karlsruhe, Germany.) test was carried out to identify the mineral phases of the sulfate-attack samples. The angular range was 3–70°, with a scanning step size of 0.02° at a current of 30 mA. The TG test was conducted by a synchronous thermal analyzer (model STA449F5, Germany Netzsch Company, Selb, Germany), at a heating rate of 5 °C/min from room temperature to 900 °C in a nitrogen atmosphere. Additionally, TG and DTG results were used to analyze the hydrated products generated in the sulfate corrosion processes.

## 3. Results and Discussion

### 3.1. Mass Change

**Figure 4:**
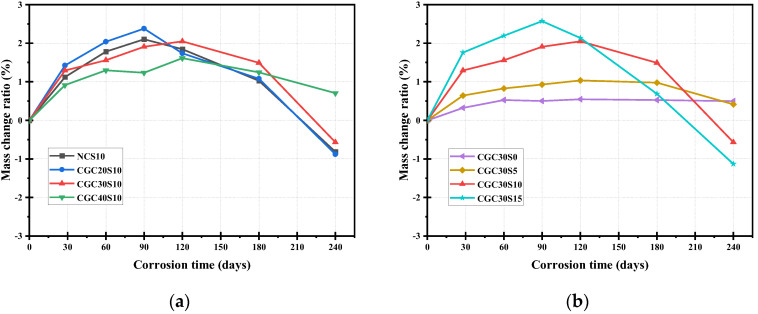
Mass change ratio of specimens at different corrosion times under sulfate attack: (**a**) different concrete; (**b**) different sulfate concentration.

Figure 4 illustrates the mass change ratio versus sulfate corrosion time of the NC and CGC specimens. In general, the mass change ratio of the specimens experienced an initial rising stage and then a falling stage with an extended corrosion time. The maximum mass of the specimens occurred at 90 or 120 d. The slight increase of the mass in the early period was mainly attributed to the expansive gypsum and ettringite generated from the reaction with diffused sulfate ions and even the deposition of sulfate salt [14]. As the sulfate corrosion continued, the expansive products accumulated in the initial pores, developing an expansion stress; when the expansion stress exceeded the tensile strength of the concrete itself, new cracks and peeling of the concrete occurred, resulting in a mass decrease in the later stage.

When comparing the sulfate resistance of NC and CGC30, it can be observed from Figure 4a that CGC30 exhibited a mass loss ratio of 0.57% after 240 corrosive days, slightly lower than the 0.88% of NC with the same mix proportion. Additionally, it is observed that the specimen with lower CGC strength showed a greater change both in the rising and falling stage. Meanwhile, the mass change ratio of CGC20S10 began to decline at an earlier corrosive age. This is the reason that a larger w/b ratio contributes to a more porous structure of CGC20, providing more pathways for the diffusion of sulfate ions to erode the concrete. The concentration of Na_2_SO_4_ solution is another vital influential factor, as depicted in Figure 4b. Under a water condition (S0), the mass change ratio of CGC30 specimens increased slightly as the age increased, without the falling stage, and the mass increase ratio was 0.49% at 240 d, which is attributed to further hydration. It can be observed that the highest mass increase of 2.5% can be found in CGC30S15-90 d, compared with 0.97% of CGC30S5-120 d and 1.91% of CGC30S15-180 d, owing to more corrosive products and deposited sulfate salt in 15% Na_2_SO_4_ solution at the preliminary stage. Additionally, the decline point appeared earlier as the sulfate concentration of the solution increased. The mass loss ratio of CGC30S15 specimens reached 1.13% at the end of the corrosive test, showing a larger decrease than that of CGC30S5 and CGC30S10. Under a relatively higher sulfate concentration attack, more cracks and spalling of CGC30 were observed at the later stage, which lead to a large reduction of mass.

### 3.2. Relative Dynamic Elastic Modulus

**Figure 5:**
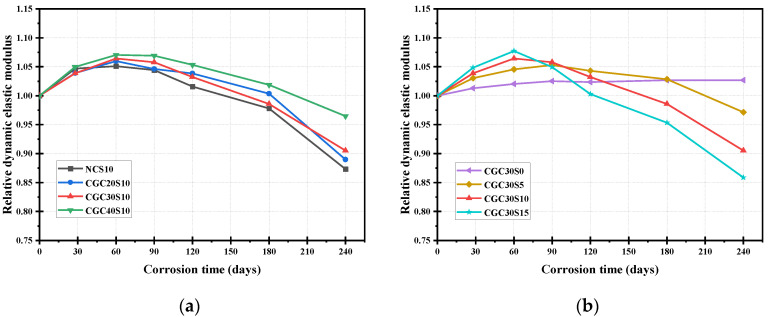
Relative dynamic elastic modulus of specimens at different corrosion times under sulfate attack. (**a**) different concrete; (**b**) different sulfate concentration.

Figure 5 presents the relative dynamic elastic modulus of the specimens with the extension of sulfate corrosion time, based on the calculated results. Similar to the law of the mass change ratio, a slight increase of relative dynamic elastic modulus was observed in the specimens under the early 90-day sulfate attack. During this time, the products, formed by further cement hydration, volcanic reaction and corrosive reaction, contributed to the compactness of the concrete, thus increasing its stiffness and dynamic elastic modulus. For a similar reason as for the mass loss, at the later stage, micro-cracks developed, and the relative dynamic elastic modulus values declined as the corrosion time increased.

It is notable from Figure 5a that the secondary hydration of coal gangue aggregates in CGC is conducive to sulfate resistance, showing a lower relative dynamic elastic value than that of NC. As for the CGC with different strengths, the relative dynamic elastic modulus of CGC40S10 increased more rapidly at the initial rising stage and declined more slowly at the falling stage as compared to CGC20S10 and CGC30S10. The final relative dynamic elastic modulus of CGC20S10, CGC30S10 and CGC40S10 was 0.89, 0.91 and 0.96, respectively. Lower deterioration of CGC40S10 was attributed to a more compact structure in a smaller w/b ratio; thus, fewer sulfate ions could penetrate the concrete. Moreover, it is concluded that the influence of Na_2_SO_4_ concentrations on the relative dynamic elastic modulus is similar to that of its influence on the mass change ratio, as shown in Figure 5b. Higher sulfate solution concentration contributed to an earlier decline and a larger descent rate of the relative dynamic elastic modulus, implying that more expansive damage was caused by the chemical reaction with more sulfate ions. As a consequence, the final relative dynamic elastic modulus of the CGC30S0, CGC30S5, CGC30S10 and CGC30S15 was 1.03, 0.97, 0.90 and 0.86, respectively. In brief, lower CGC strength and higher sulfate solution concentrations adversely affected the dynamic elastic modulus of CGC.

### 3.3. Compressive Strength

**Figure 6:**
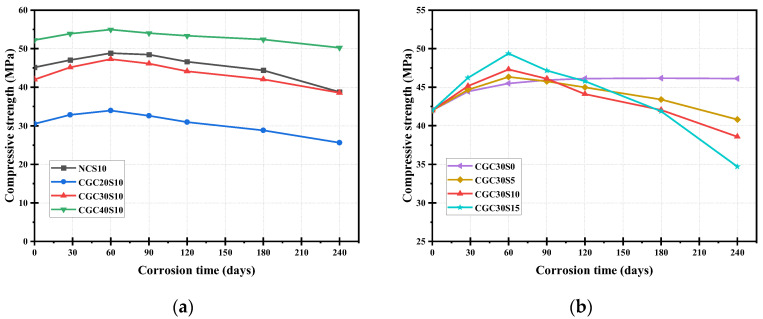
Compressive strength of specimens at different corrosion times under sulfate attack: (**a**) different concrete; (**b**) different sulfate concentration.

**Figure 7:**
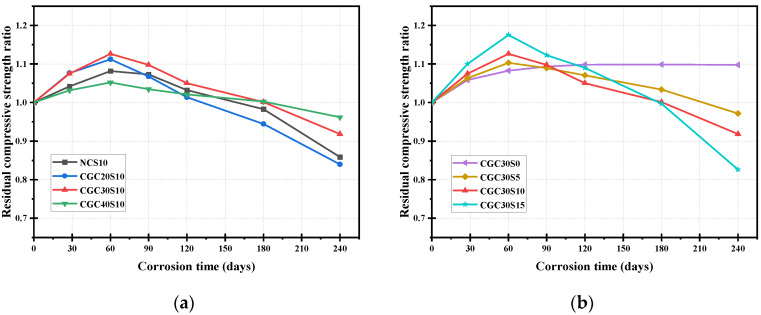
Residual compressive strength ratio of specimens at different corrosion times under sulfate attack: (**a**) different concrete; (**b**) different sulfate concentration.

Figure 6 and Figure 7 illustrate the evolution of compressive strength and residual ratio for sulfate-attacked specimens in each group. Except for CGC30S0, the compressive strength of sulfate-attacked concrete specimens presented a similar trend in general: it first increased and then declined with the corrosion time. The products generated by further cement hydration and sulfate corrosive reaction jointly filled the pores and densified the structure of the concrete at the initial stage, leading to a slight increase in compressive strength. However, more expansive products from the sulfate corrosive reaction caused many micro-cracks in internal concrete as the time increased, and thus, the strength started to decrease from about 60–90 d until the end of the sulfate attack at 240 d.

Before exposure to the sulfate attack, as shown in Figure 6a, the compressive strength of CGC30 was 42.01 MPa, about 7.0% lower than that of NC (45.15 Mpa) with the same mix proportion; the reason for this is that the fine and coarse aggregates in CGC30 are totally replaced by coal gangue, and the inherent properties of coal gangue are poorer than that of natural aggregates. It is noteworthy from Figure 7a that the strength increase slope of CGC30 is larger than that of NC under a 10% sulfate solution attack. This phenomenon may be attributed to the following reason: the pozzolanic activity of coal gangue aggregates plays a positive role in further cement hydration, and thus, more increase in strength [19,31]. Additionally, it is shown in Figure 7a that the residual strength ratio of CGC30S10-240 d and NCS10-240 d is 0.92 and 0.86, respectively, implying that the sulfate resistance of CGC may be superior to that of NC. As shown in Figure 6a and Figure 7a, in the initial rising stage, it is observed that the compressive strength of CGC20S10 and CGC30S10 increased by 11.2% and 12.6%, respectively, owing to further cement hydration and corrosive reaction with diffused sulfate ions. In contrast, the strength increase of CGC40S10 at the early stage was small, only 2.73 Mpa accounting for 5.2%; the reason for this is that CGC40 with a lower w/b ratio has fewer pores to diffuse the sulfate ions, and thus, the capacity of deposited products is restricted, resulting in a smaller increase in compressive strength. Moreover, the strength increase ratio of CGC40S10 was also limited by the lower strength of coal gangue aggregates. With the extension of corrosion time, the compressive strength began to decline and the residual ratio of CGC20S10-240 d, CGC30S10-240 d and CGC40S10-240 d was 0.83, 0.92 and 0.96, respectively. Under the same sulfate condition, the lower the CGC strength, the more severe the deterioration. The main reason could be attributed to the fact that the CGC with a higher w/b ratio has more initial pores and paths allowing the sulfate ions to penetrate the interior and generate expansive products, which may induce more new micro-cracks in the later dropping stage.

It is clearly seen from Figure 7b that the compressive strength of CGC30S0, exposed to water solution, showed an increasing tendency before 120 d, and then tended to be stable, mainly attributed to further cement hydration and second hydration reaction between coal gangue and calcium hydroxide (CH) [8]. Noticeably, under 5%, 10% and 15% sulfate solution attack, the compressive strength of specimens first increased and then decreased. In addition, the compressive strength of CGC30S5-60 d, CGC30S10-60 d and CGC30S15-60 d was 46.34 Mpa, 47.31 Mpa and 49.26 Mpa, respectively, indicating that the strength rising slope increased as sulfate concentration increased. In a 15% sulfate solution, more diffused sulfate ions participate in the reaction to form expansive products, first filling pores to improve the strength and then inducing cracks to damage the CGC. The residual strength ratio of CGC30S5-240 d, CGC30S10-240 d and CGC30S15-240 d was 0.97, 0.92 and 0.83, respectively. Hence, it can be concluded that a higher sulfate solution concentration contributes to a larger variation range between the highest and lowest strength in the sulfate corrosive process. This is mainly for the reason that a higher sulfate concentration solution could promote the diffusion of more sulfate ions into the concrete, accelerating the degradation of concrete.

### 3.4. Distribution of Sulfate Ions

**Figure 8:**
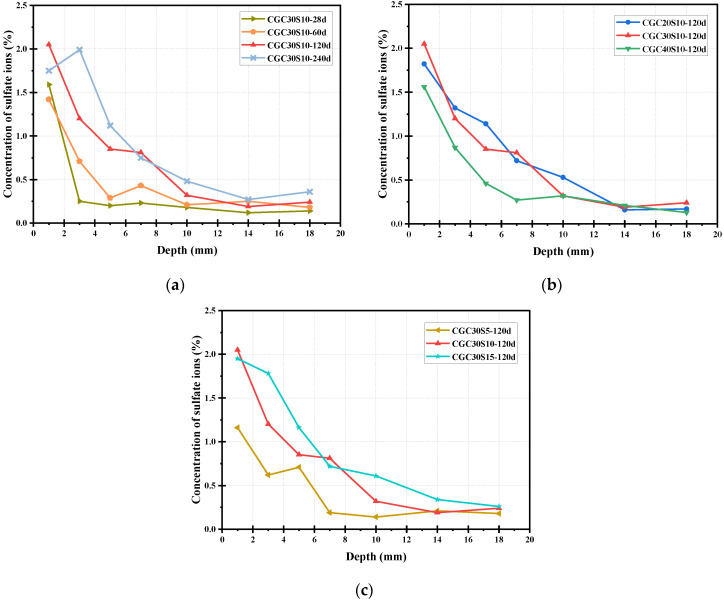
Distribution of sulfate ions in different sulfate-attacked concrete: (**a**) corrosion time; (**b**) CGC strength; (**c**) sulfate concentration.

Figure 8 depicts the concentration of sulfate ions at different depths in different sulfate-attacked concrete. For instance, it should be noted that the $\omega \left({\mathrm{SO}}_{4}^{2-}\right)$ results of 0–2 mm powder samples were used to represent the sulfate ion content at a depth of 1 mm from the exposed surface. It can be clearly seen from Figure 8 that the distribution of sulfate ions generally exhibited a similar trend, where the concentration of sulfate ions first declined gradually and then almost kept stable with the increase of depth. Noticeably, the stable value of $\omega \left({\mathrm{SO}}_{4}^{2-}\right)$ is not exactly zero because there may be sulfate substances in the internal cementitious materials [23].

As shown in Figure 8a, in the dropping stage, it is observed that the slope of sulfate ion content was decreasing more slowly with attack time, and the largest decrease of the slope appeared in CGC3010-28 d due to the short time for the diffusion of sulfate ions. On the other hand, a longer sulfate corrosion time of 240 d contributed to a higher concentration of sulfate ions at the same depth when compared with other groups. Furthermore, it is indicated that the sulfate corrosive depth of CGC30S10-28 d was about 3 mm, whereas that of CGC30S10-240 d was 14 mm, implying that the sulfate corrosive depth increased as the corrosion time increased. Correspondingly, it is concluded that the longer the sulfate corrosion time was, the more severe the deterioration was.

When compared with GC20S10-120 d and CGC30S10-120 d, as shown in Figure 8b, the $\omega \left({\mathrm{SO}}_{4}^{2-}\right)$ of CGC40S10-120 d was lower at the same depth, and its final sulfate penetration depth was also lower, which implied a superior sulfate resistance. This may be attributed to the dense structure and fewer pores in CGC40, hindering the progress of sulfate attack. Moreover, the sulfate penetration depth in CGC40S10-120 d is also lower. It is well accepted that the diffusion of sulfate ions is motivated by the concentration gradient between the internal and external environment, according to Fick’s law. As expected, in Figure 8c, compared with CGC30S5-120 d and CGC30S10-120 d, the sulfate ions of CGC30S15-120 d penetrated more and deeper when exposed to a higher concentration of sulfate solutions. It can be clearly observed that the sulfate corrosive depth of CGC30S5-120 d, CGC30S10-120 d and CGC30S15-120 d was about 7 mm, 10 mm and 14 mm, respectively. Moreover, CGC exposed to a higher concentration of sulfate attack would exhibit a relatively low compressive strength as discussed before, indicating a more severe degradation after corrosion.

## 4. Micro-Mechanism of Sulfate-Attacked CGC

The physical and mechanical properties and durability of concrete are closely related to its microstructural and mineral characteristics. Differently from NC with inert river sand and natural gravel, it is demonstrated that the active SiO_2_ and Al_2_O_3_ of coal gangue fine aggregates in CGC participate in the secondary hydration reaction consuming the CH crystals to form calcium silicate hydrate (C-S-H) gels and ettringite (Aft) crystals, which is conducive to filling the initial pores and improve the strength of CGC [32,33]. Under sulfate attack, the sulfate ions diffuse into the concrete and react chemically with hydration products. Therefore, it is necessary to reveal the sulfate corrosive mechanism of CGC through SEM, XRD and TG tests.

### 4.1. SEM Analysis

**Figure 9:**
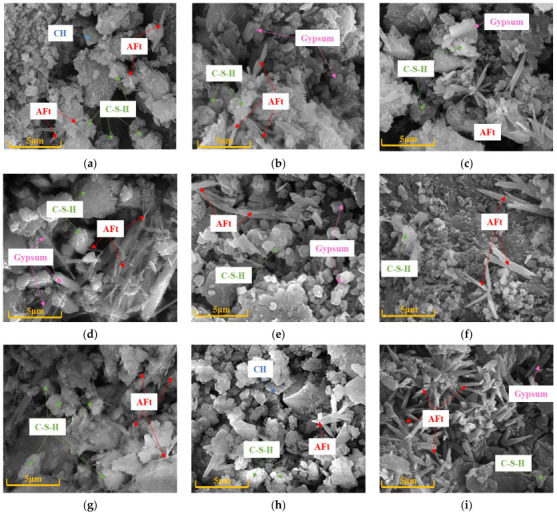
SEM images of different sulfate-attacked CGC specimens: (**a**) CGC30S10-28 d; (**b**) CGC30S10-60 d; (**c**) CGC30S10-120 d; (**d**) CGC30S10-240 d; (**e**) CGC20S10-120 d; (**f**) CGC40S10-120 d; (**g**) CGC30S0-120 d; (**h**) CGC30S5-120 d; (**i**) CGC30S15-120 d.

Figure 9 illustrates the micromorphology of selected CGC specimens under different sulfate conditions. Obviously, the microstructure was significantly affected by the considered variables in the aspects of amorphous C-S-H gel, needle-like Aft crystal, plate-like CH crystal, and short columnar gypsum [15,27]. With the extension of sulfate corrosion time, it can be observed from Figure 9a–d that the plate-like CH crystals gradually disappeared, while needle-like ettringite and short columnar gypsum developed. This phenomenon occurs for the reason that the invading sulfate ions reacted with the hydrated products of CGC and formed expansive products, including the ettringite and gypsum, as demonstrated in the literature [22,23,24]. Moreover, it is worth noting that the number of ettringite crystals increased, and the shape grew gradually from slender needle-like to rod-like as the sulfate corrosion time increased. In the early corrosive stage, the newly generated Aft and gypsum tended to play a filling and refining impact on the pores to improve the properties of concrete, and obviously in Figure 9b, the microstructure of CGC30S10-60 d became denser, providing the reason for the optimal compressive strength at 60 d. However, the expansive Aft and gypsum, accumulated in the pores, could produce pressure on the walls of the pores. As shown in Figure 9c,d, due to the large amount of Aft crystals and gypsum, when the expansion stress surpassed the tensile strength of the concrete itself, new cracks were generated, leading to a decrease in mechanical properties. Moreover, the new cracks provided more paths to diffuse sulfate ions to damage the CGC. In particular, it can be observed from Figure 9d that the C-S-H gels were gradually coarsened, suggesting the dissolution of calcium. This is mainly attributed to the fact that the exhausting CH crystals caused the decrease of pH value in concrete with the extension of sulfate corrosion [34], and thus, the decalcification of C-S-H occurred for the reaction between calcium ions and sulfate ions, producing more expansive gypsum and Aft to damage CGC.

When comparing the specimens of different CGC strengths, as shown in Figure 9c,e,f, the overall morphology of CGC40S10 was compact, while the microstructure of CGC20S10 and CGC30S10 was relatively loose after sulfate exposure of 120 d. This is because CGC40 with a lower w/b ratio had fewer initial pores and cracks, and thus, fewer sulfate ions could diffuse into the CGC to form expansive products, leading to a lower deterioration of CGC40 under sulfate attack. In addition, it can be clearly observed from Figure 9e that the micro-cracks were generated in CGC20 due to its loose structure and expansion of accumulated Aft and gypsum in many pores [31], which resulted in the larger decrease of compressive strength in the dropping stage. Notably, without the sulfate attack, the morphology of CGC30S0-120 d in Figure 9g was mainly C-S-H gels, CH and Aft crystals, consistent with the major hydrated products of CGC. It is clarified that the sulfate corrosive reaction consumed CH crystals to form gypsum and Aft. Thus, there are still CH crystals appearing in CGC30S5-120 d due to the less penetrated sulfate ions under lower sulfate solution concentration. Moreover, as shown in Figure 9i, no CH crystals, more ettringite crystals and even cracks were observed in CGC30S15-120 d, implying the worst deterioration when exposed to the 15% sulfate solution corrosion. It can be concluded from Figure 9c,g–i that, as expected, with the increase of sulfate solution concentration, the amount of CH crystals declined, while the amount of expansive products and cracks increased at the same corrosion time of 120 d.

### 4.2. XRD Analysis

**Figure 10:**
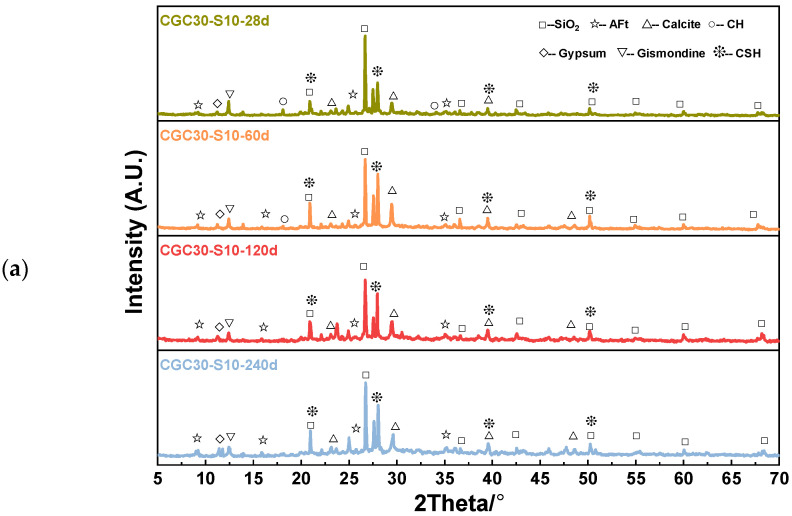

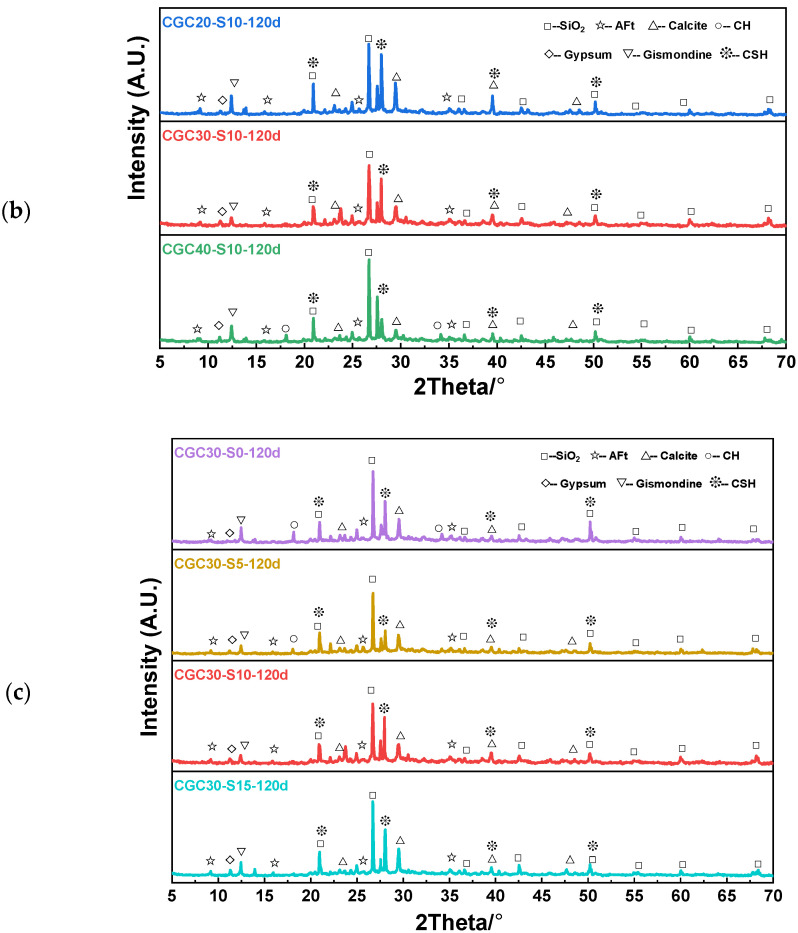
XRD patterns of different sulfate-attacked CGC specimens: (**a**) corrosion time; (**b**) CGC strength grade and (**c**) sulfate concentration.

The XRD results of different sulfate-attacked CGC specimens are presented in Figure 10. In general, the considered variables, including corrosion time, CGC strength grade and sulfate concentration, had a significant impact on the diffraction peak intensity of various phases. The XRD spectra indicated that the major phases of CGC are CH crystals, C-S-H gels, gypsum and AFt crystals after exposure to sulfate solution spray, in accordance with the SEM results. Moreover, it should be noted that gismondine is the secondary hydration product, generated from the reaction between CH crystals and active Al_2_O_3_ and SiO_2_ in CGC [5].

As shown in Figure 10a, with the extension of sulfate corrosion time, the diffraction peak of CH (d = 4.93 and 2.64 Å) became less intense and even disappeared, and simultaneously, the intensity of the ettringite peak (d = 9.82, 5.54 and 2.56 Å) and gypsum peak (d = 7.69 Å) increased, where the value of d is the interplanar spacing according to the Bragg equation. The reason for this is that the CH crystals participated in the reaction with the diffused sulfate ions to form gypsum, and then the gypsum was consumed to generate AFt crystals. It is noteworthy that the increased AFt crystals from sulfate corrosive reaction and C-S-H from further cement hydration were observed in CGC30S10-60 d, jointly conducive to the improvement of compressive strength. In contrast, as the corrosion time grew to 120 d and 240 d, it is implied that the C-S-H were consumed with the process of sulfate corrosion. Moreover, too much expansive AFt and gypsum in CGC30S10-120 d and CGC30S10-240 d had a negative effect on the strength of CGC due to its expansive stress on the walls of pores, causing new cracks and pores to accelerate the process of sulfate attack. This phenomenon was consistent with the results of compressive strength. Furthermore, it is interesting that the intensity of the gismondine peak (d = 7.37 Å) decreased as the sulfate corrosion progressed. This may be attributed to the fact that more hydrated calcium aluminate was needed to form the ettringite in the presence of more gypsum, and thus the dealuminizing effect on gismondine caused the decrease of gismondine.

As for the effect of CGC strength grade, it is observed from Figure 10b that the CH peaks disappeared in the specimens of CGC20S10-120 d and CGC30S10-120 d due to its consumption in the sulfate corrosive reaction. Nevertheless, the CGC40 had an initial dense structure with low porosity, which was adverse to diffusing sulfate ions into concrete, and consequently, the CH crystals were consumed less, and the AFt crystals were generated less after 120 d corrosion of 10% sulfate solution. Thus, the diffraction peak of CH in CGC40S10-120 d was observed in Figure 10b. Furthermore, it is demonstrated in Figure 10c that the intensity of the CH peak declined as the concentration of sulfate solutions increased. Notably, the CH peak of CGC30S0-120 d was relatively intense, whereas, in the specimens of CGC30S10-120 d and CGC30S15-120 d, the diffraction peaks of CH crystals were barely visible, owing to the sulfate corrosive reaction between more diffused sulfate ions and CH crystals in a higher concentration of sulfate solution. It is also observed from Figure 10c that the intensity of the gypsum peak was highest in CGC30S15-120 d among four specimens with different sulfate concentrations of the solution, indicating that gypsum, rather than ettringite, became the dominant corrosive product in a high concentration of Na_2_SO_4_ solution, due to the rapid consumption of aluminate during the long corrosive process. The volume of gypsum expanded to 2.2 times the original, which led to the generation of new cracks and severe deterioration in CGC exposed to a higher concentration of sulfate solution [10].

### 4.3. TG-DTG Analysis

**Figure 11:**
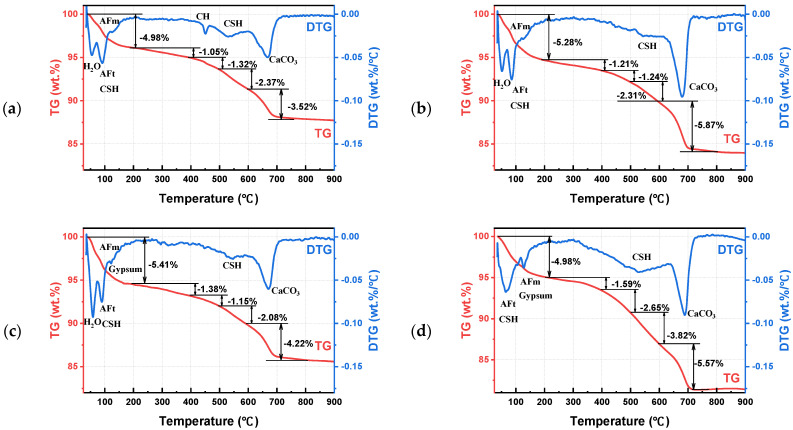

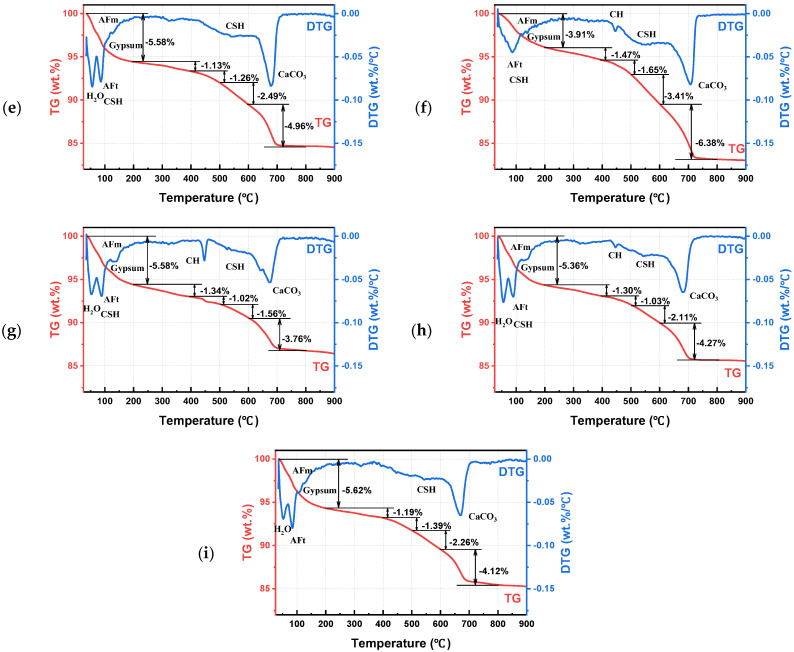
TG and DTG curves of different sulfate-attacked CGC specimens: (**a**) CGCS10-28 d; (**b**) CGCS10-60 d; (**c**) CGC30S10-120 d; (**d**) CGC30S10-240 d; (**e**) CGC20S10-120 d; (**f**) CGC40S10-120 d; (**g**) CGC30S0-120 d; (**h**) CGC30S5-120 d; (**i**) CGC30S15-120 d.

TG-DTG curves were used to characterize the mass change of hydrated and corrosive products at the corresponding temperature in sulfate-attacked CGC specimens. It should be noted that the DTG results are the first-order differential of TG data, reflecting the mass loss rate during thermal decomposition. As depicted in Figure 11, after the sulfate corrosion, the TG mass loss trend of different specimens was similar, but the DTG curves varied in the mass loss rate, width and intensity of the peak. It is clearly annotated that the peaks emerging at about 90 °C, 440 °C and 670 °C corresponded to the products of AFt crystals, CH crystals and CaCO_3_, respectively [5,35]. Moreover, the gypsum peaks took place at approximately 120 °C, which may be attributed to the escape of water. Additionally, the C-S-H is identified at temperatures of about 100 °C and 540 °C.

In agreement with the results of SEM and XRD, it is manifested in Figure 11a–d that the CH peak at about 440 °C appeared in CGC30S10-28 d, however, as the corrosion time extended to 120 d and 240 d, the CH peak gradually disappeared due to its consumption in a sulfate corrosive reaction. Additionally, the absolute value of AFt crystals in DTG curves increased with the extension of sulfate corrosion time, indicating that more ettringite was generated from more diffused sulfate ions. In particular, it can be observed from Figure 11b that the mass loss during the temperature of 20~200 °C increased in CGC30S10-60 d due to further cement hydration and secondary hydration with coal gangue. Moreover, the amount of AFt crystals increased appropriately at the early stage, filling the pores of CGC, which jointly contributes to the superior properties of CGC30S10-60 d as analyzed before. In addition, it is shown in Figure 11d that the mass loss ratio in CGC30S10-240 d during the whole process of thermal decomposition is 18.61%, larger than that of CGC30S10-28 d, CGC30S10-60 d and CGC30S10-120 d. The reason may be the fact that a more serious degradation in CGC30S10-240 d leads to more corrosive products of AFt and gypsum.

When it comes to the effect of CGC strength grade on sulfate corrosion, it can be seen from Figure 11c,e,f that the absolute value of AFt peak increased as the CGC strength decreased, similar to the analysis in XRD patterns. Especially due to the dense structure and fewer pores of CGC40, it is difficult for sulfate ions to penetrate the CGC and participate in sulfate corrosive reaction, and thus, less consumption of CH led to the appearance of CH peaks and relatively low degradation in CGC40S10-120 d. It is also indicated that the mass loss percentage ranging from 400 °C to 500 °C in CGC40S10-120 d is 1.65%, larger than that of CGC20S10-120 d (1.26%) and CGC30S10-120 d (1.15%). Moreover, it is also observed in Figure 11c,g–i that the intensity of the CH peak decreased as the concentration of sulfate solutions increased, owing to its consumption in a higher concentration of sulfate solution. Compared with CGC30S5-120 d and CGC30S10-120 d, the mass loss rate of AFt and gypsum peak in CGC30S15-120 d is larger, indicating that a higher concentration of sulfate solution contributed to more generated expansive products to damage CGC, and thus, severe deterioration was observed in compressive strength. In addition, when carefully comparing the DTG curves between Figure 11h,i, it is seen that the peak of C-S-H appearing at around 540 °C in CGCS5-120 d is slightly higher than that in CGCS15-120 d, further proving its decalcification under the condition of many sulfate ions. As discussed above, it can be concluded that more diffused sulfate ions could lead to more expansive products in a chemical reaction, with a lower CGC strength grade, higher sulfate solution concentration and longer corrosive age.

### 4.4. Microscopic Sulfate Corrosion Model

**Figure 12:**
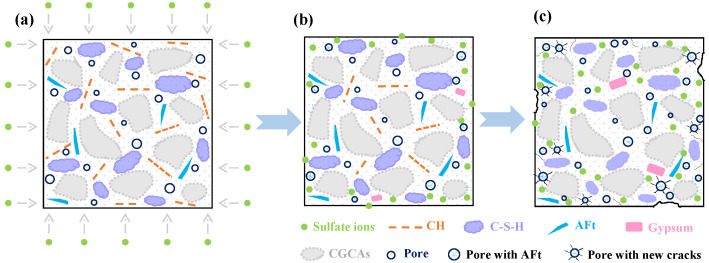
Schematic diagram of sulfate corrosion model: (**a**) ingress of sulfate ions; (**b**) gradual penetration of sulfate ions; (**c**) damage of CGC.

Based on the microscopic analysis of SEM, XRD and TG, it is indicated that chemical attack is the key cause of deterioration in CGC when exposed to an underground sulfate-rich environment. Combining the hydration and the sulfate corrosion mechanism analyzed before, the sulfate corrosion model of CGC is mainly summarized as three stages: the ingress of SO_4_^2−^, the gradual penetration of sulfate ions and the damage of CGC, as depicted in Figure 12.

In the first stage, it can be seen from Figure 12a that the surrounding sulfate ions started to enter into the CGC when the CGC with preliminary hydration was placed in the sulfate environment. It is worth noting that the hydration process of CGC was slightly different from that of NC [5]. Except for the normal hydration products of cement, the partial CH crystals participated in a secondary hydration reaction to form C-S-H gels and AFt crystals due to the activated Al_2_O_3_ and SiO_2_ of coal gangue in CGC. Thus, compared with NC, the structure of CGC may be denser with fewer CH crystals, more C-S-H gels and AFt crystals.

With the ingress of sulfate ions through the original pores, a large amount of SO_4_^2−^ was attached to the surface of CGC and reacted with the CH crystals. As shown in Figure 12b, the AFt crystals and gypsum were generated from the sulfate corrosive reactions consuming SO_4_^2−^ and CH crystals, and the reactions were mainly expressed in the following three aspects:

(6) $$CH+SO_{4}^{2-}+2H\to C\bar{S}H_{2}+2OH^{-},$$

(7) $$C_{4}A\bar{S}H_{12}+2C\bar{S}H_{2}+16H\to C_{6}A{\bar{S}}_{3}H_{32},$$

(8) $$C_{4}AH_{13}+3SO_{4}^{2-}+2CH+20H\to C_{6}A{\bar{S}}_{3}H_{32}+6OH^{-}\;$$

It should be noted that “C”, “H”, “$\bar{S}$” and “A” in the reactions are the abbreviations of “CaO,” “H_2_O,” “SO_3_” and “Al_2_O_3_” chemical formulas, respectively. Obviously, the CH crystals were gradually consumed with the diffusing sulfate ions. In addition, it is indicated that the formed gypsum ($C\bar{S}H_{2}$) was soon involved in the generation of AFt crystals, and thus, little gypsum could be left in the initial stage. More importantly, the generated AFt crystals ($C_{6}A{\bar{S}}_{3}H_{32})$ could gradually fill the pores and interfacial transition zone, which is conducive to the improvement of compactness and strength in CGC during the early sulfate corrosion.

Nevertheless, the gypsum and AFt crystals produced expansive stress on the walls of pores due to their expansive properties. As the sulfate corrosive reaction proceeded, the expansive pressure increased continuously, and when it exceeded the tensile strength of CGC, new micro-pores and cracks appeared with the accumulated internal damage. It can be observed from Figure 12c that the new pores and cracks provided more channels for the penetration of sulfate ions and more space for the formation of expansive products, accelerating the damage of CGC, such as cracking and spalling. Thus, the mass and compressive strength are significantly reduced. In the later process of corrosion, the CH crystals were almost consumed completely, and the decalcification of C-S-H occurred to balance the pH value of the internal CGC. Meanwhile, a large amount of generated AFt crystals played a negative effect on the properties, contributing to the severe degradation of CGC in a sulfate-rich environment.

## 5. Conclusions

In this paper, to expand the utilization of coal gangue in an underground sulfate-rich environment, the deterioration process of sulfate-attacked CGC was elucidated from the aspect of macro properties and micro mechanisms. According to the test results and analysis, the following conclusions can be drawn:

The residual compressive strength ratio of CGC30S10-240 d was 0.92, higher than the 0.86 of NCS10-240 d. It is indicated that the volcanic activity of SiO_2_ and Al_2_O_3_ in coal gangue plays a positive role in a denser structure and a superior sulfate resistance of CGC when compared with NC.

A lower CGC strength leads to a larger mass loss ratio, a lower relative dynamic elastic modulus and a lower residual compressive strength ratio of CGC after a 240 d sulfate attack. Moreover, the sulfate concentration of the solution is a key factor affecting the performance of CGC, and higher sulfate concentration tends to cause more severe degradation of sulfate-attacked CGC.

For sulfate-attacked CGC specimens, the concentration of diffused sulfate ions declined and then almost kept stable with increasing depth from the surface. Higher sulfate concentration and longer corrosion time contribute to a larger sulfate corrosion depth of CGC, indicating a more serious degradation. In particular, higher strength of CGC contributes to lower permeability and better sulfate resistance.

Chemical attack is dominant in the deterioration of sulfate-attacked CGC. Except for the secondary hydration reaction between coal gangue and CH, the diffused sulfate ions also participate in the reaction with CH to form short columnar gypsum and needle-like ettringite, which has a filling effect on the internal pores at the early stage. Nevertheless, as the corrosion time increased, a large number of corrosion products caused expansive pressure and initiated cracks to damage the CGC.

A microscopic sulfate corrosion model is proposed, which is mainly summarized as three stages: the ingress of SO_4_^2−^, the gradual penetration of sulfate ions and the damage of CGC. Particularly, the decalcification of C-S-H gels and the dealumination of gismondine were inferred in sulfate-attacked CGC.

## Figures and Tables

**Table 1:** Chemical composition of raw coal gangue (mass fraction).

Chemical Composition	SiO_2_	Al_2_O_3_	CO_3_	Fe_2_O_3_	K_2_O	CaO	Na_2_O	MgO	Others
/%	61.69	19.11	5.83	4.16	3.04	2.35	2.28	0.64	0.9

**Table 2:** The basic properties of fine and coarse aggregate.

Material Characteristics	Particle Size Distribution	Bulk Density (kg/m^3^)	Water Absorption (%)	Crushing Value (%)	Fineness Modulus
Coal gangue coarse aggregate	4.75–20 mm	1222.18	1.34	8	/
Natural gravel	4.75–20 mm	1376.75	0.42	1.2	/
Coal gangue fine aggregate	0.15–4.75 mm	1474.62	3.06	/	3.20
River sand	0.15–4.75 mm	1562.39	0.93	/	3.16

**Table 3:** Detailed test design of factor level.

Series	Concrete Strength	Sulfate Concentration	Corrosion Time
1	CGC20, CGC30, CGC40, NC	S10	28 d, 60 d, 90 d, 120 d, 180 d, 240 d
2	CGC30	S0, S5, S10, S15	28 d, 60 d, 90 d, 120 d, 180 d, 240 d

**Table 4:** Mix proportion of concrete (kg/m^3^).

Group	Cement	Water	Coal Gangue Fine Aggregate	River Sand	Coal Gangue Coarse Aggregate	Natural Gravel	Superplasticizer
CGC20	271.4	190	690.7	/	1227.9	/	0.3%
CGC30	345.5	190	664	/	1180.5	/	0.4%
CGC40	475	190	617	/	1098	/	0.5%
NC	345.5	190	/	664	/	1180.5	0.2%

## Data Availability

Not applicable.
